# Collagen-Derived Dipeptide Pro-Hyp Enhanced ATDC5 Chondrocyte Differentiation under Hypoxic Conditions

**DOI:** 10.3390/molecules28124664

**Published:** 2023-06-09

**Authors:** Yoshifumi Kimira, Takahiro Sato, Mayu Sakamoto, Yoshihiro Osawa, Hiroshi Mano

**Affiliations:** Department of Clinical Dietetics and Human Nutrition, Faculty of Pharmacy and Pharmaceutical Sciences, Josai University, 1-1 Keyakidai, Sakado-shi 350-0295, Saitama, Japan

**Keywords:** collagen-derived peptide, prolyl-hydroxyproline, chondrocyte differentiation, physiological hypoxic condition, cartilage, extracellular matrix

## Abstract

Chondrocytes are surrounded by a lower oxygen environment than other well-vascularized tissues with higher oxygenation levels. Prolyl-hydroxyproline (Pro-Hyp), one of the final collagen-derived peptides, has been previously reported to be involved in the early stages of chondrocyte differentiation. However, whether Pro-Hyp can alter chondrocyte differentiation under physiological hypoxic conditions is still unclear. This study aimed to investigate whether Pro-Hyp affects the differentiation of ATDC5 chondrogenic cells under hypoxic conditions. The addition of Pro-Hyp resulted in an approximately 18-fold increase in the glycosaminoglycan staining area compared to the control group under hypoxic conditions. Moreover, Pro-Hyp treatment significantly upregulated the expression of SOX9, Col2a1, Aggrecan, and MMP13 in chondrocytes cultured under hypoxic conditions. These results demonstrate that Pro-Hyp strongly promotes the early differentiation of chondrocytes under physiological hypoxic conditions. Therefore, Pro-Hyp, a bioactive peptide produced during collagen metabolism, may function as a remodeling factor or extracellular matrix remodeling signal that regulates chondrocyte differentiation in hypoxic cartilage.

## 1. Introduction

The extracellular matrix (ECM) of tissues is essential for cell adhesion and function, and collagen is a critical component for maintaining ECM homeostasis [1,2]. Collagen turnover produces bioactive collagen peptides (CP) from enzymatic degradation [3,4]. There are different types of collagen molecules: type I collagen is the primary collagen in skin, bone, and tendons; type II collagen is the primary collagen in cartilage. Collagen contains at least one glycine (Gly)-X-Y common repeat domain, where X and Y are predominantly proline (Pro) and hydroxyproline (Hyp), respectively, and many bioactive peptides derived from collagen contain Hyp in their sequences [5,6]. One of the final collagen metabolites, prolyl-hydroxyproline (Pro-Hyp), has been shown to increase the number of fibroblasts migrating from the mouse skin and promote the differentiation of osteoblasts, tendon cells, and chondrocytes [7,8,9,10].

Chondrocytes are the only cell type existing in cartilage and maintain the equilibrium of ECM and cartilage homeostasis by producing cartilage matrix proteins such as type II collagen α1 (Col2a1) and proteoglycans including Aggrecan (Acan) [11,12]. The transcription of Col2a1 and Aggrecan is strongly induced by sex-determining region Y-box 9 (Sox9) [13], while matrix metalloproteinase 13 (Mmp13) plays essential roles in cartilage degeneration [14]. Cartilage chondrocytes surround a hypoxic environment, unlike other well-vascularized tissues [15,16]. The physiological oxygen concentration in the cartilage’s deepest layer is about 1% [17]. Therefore, chondrocyte differentiation may be influenced by Pro-Hyp under physiological hypoxic conditions.

Previous studies have reported that Pro-Hyp is involved in the early stages of chondrocyte differentiation [10], but its effects under physiological hypoxic conditions remain unclear. In this study, Pro-Hyp strongly enhanced early chondrogenic differentiation under physiological hypoxic conditions. 

## 2. Results and Discussion

### 2.1. Pro-Hyp Regulates Chondrogenic ATDC5 Cells Differentiation in Hypoxic Condition 

**Figure 1:**
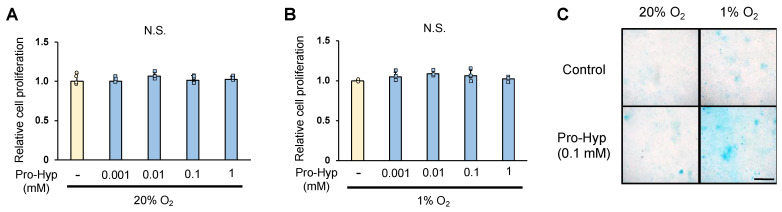
ADTC5 chondrocytes were seeded at 3 × 10^3^ cells/well on a 96-well plate and cultured for 24 h at 37 °C. After treatment with a range of Pro-Hyp (0, 0.001, 0.01, 0.1, and 1 mmol/L) cultured in normoxic condition (20% oxygen) or hypoxic condition (1% oxygen) for 24 h (**A**,**B**). The ATDC5 cells were cultured in either normoxic or hypoxic conditions, with or without Pro-Hyp (0.1 mmol/L), for 21 days. Scale bar, 500 μm. (**C**). Data are expressed as means ± SD. Dots represent individual samples. N.S.: no significant differences.

We analyzed whether Pro-Hyp affects the proliferation and differentiation of ATDC5 cells. The proliferation of chondrocytes was quantified by WST assay on day 1 of culture; Pro-Hyp addition did not affect ATDC5 cell proliferation under either normoxic conditions (20% oxygen) or hypoxic conditions (1% oxygen) (Figure 1A,B). When the glycosaminoglycan staining area was compared with Alcian blue staining as an index of chondrocyte differentiation, ATDC5 cells cultured for 21 days under normoxic conditions showed approximately 1.4-fold larger glycosaminoglycan staining area with Pro-Hyp addition compared to the control. ATDC5 cells cultured under hypoxic conditions for 21 days showed an approximately 1.2-fold increase in glycosaminoglycan staining area compared to those cultured under normoxic conditions. The addition of Pro-Hyp showed an approximately 18-fold increase in glycosaminoglycan staining area compared to the control group under hypoxic conditions (Figure 1C). These results indicate that Pro-Hyp does not affect chondrocyte proliferation under hypoxic conditions, but strongly promotes chondrocyte differentiation.

### 2.2. Pro-Hyp Further Upregulates Chondrogenesis-Specific Genes and ECM Remodeling Regulators under Hypoxic Conditions Compared to Normoxic Conditions

**Figure 2:**
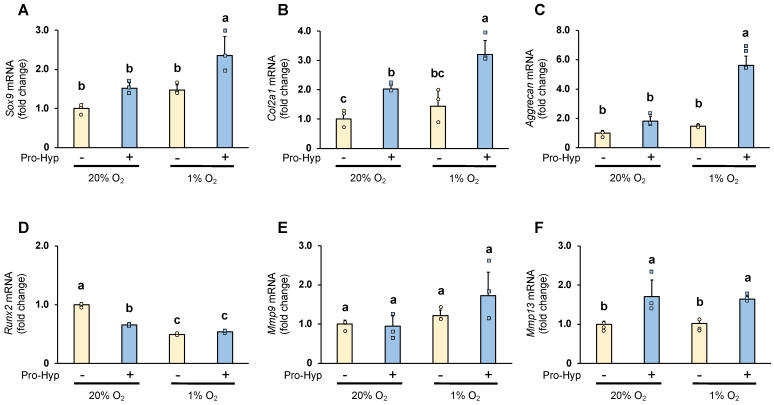
ATDC5 cells were seeded in a 35 mm dish at a density of 9 × 104 cells/well and cultured in either normoxic condition (20% oxygen) or hypoxic condition (1% oxygen) for four days. The cells were treated with or without Pro-Hyp (0.1 mmol/L) for 8 h before harvest. mRNA expression of (**A**) *Sox9*, (**B**) *Col2a1*, (**C**) *Aggrecan*, (**D**) *Runx2*, (**E**) *Mmp9* and (**F**) *Mmp13* in ATDC5 cells. Data are expressed as means ± SD. Dots represent individual samples. Different letters above the boxes indicate significant differences. *p* < 0.05.

The effect of Pro-Hyp on the expression of chondrogenesis-specific genes and ECM regulators under hypoxic conditions was analyzed by qRT-PCR. Under normoxic conditions, compared to the control, the cells cultured in the medium containing Pro-Hyp exhibited approximately 1.5-, 2-, and 3-fold increases in *Sox9*, *Col2a1*, and *Aggrecan* mRNA levels, respectively (Figure 2A–C). *Sox9*, *Col2a1*, and *Aggrecan* mRNA expression were significantly upregulated by Pro-Hyp treatment and further upregulated by culture under hypoxic conditions. We next evaluated Runx2 expression to determine whether Pro-hyp affects a vital regulator of chondrocyte hypertrophy. Runx2 mRNA expression was reduced by 40% when cultured under hypoxic conditions. Then, adding Pro-hyp under normoxic conditions dramatically reduced *Runx2* mRNA expression, but this effect was counteracted under hypoxic conditions (Figure 2D). 

Sox9 is an important chondrogenic transcription factor that maintains the early stages of chondrogenic differentiation and embryonic chondrogenesis by promoting the expression of Col2a1 and Aggrecan [13]. Runx2 is also a factor that regulates chondrogenic differentiation and promotes hypertrophy [18]. Our results suggest that Pro-hyp regulates early chondrocyte differentiation by strongly upregulating Sox9 expression without affecting Runx2 expression in chondrocytes under hypoxic conditions.

Degrading the extracellular matrix by matrix metalloproteinases (MMPs) secreted by the chondrocytes is vital for cartilage formation [19,20]. Under hypoxic conditions, *Mmp9* and *Mmp13* mRNA was unchanged compared to the control group under normoxic conditions. However, the effect of Pro-Hyp on *Mmp9* mRNA expression under hypoxic conditions was increased 1.4-fold compared to the control group. Furthermore, Pro-Hyp significantly increased *Mmp13* mRNA expression under both normoxic and hypoxic conditions (Figure 2E,F). MMP13 plays a vital role in skeletogenesis, as MMP13 deficiency may disrupt proper collagenase-mediated cleavage that usually occurs in the growth plate and primary ossification centers, resulting in delayed endochondral ossification [21]. It has also been reported that degraded fragments of type II collagen function as MMP activators that regulate the catabolic pathway of matrix turnover by stimulating the expression of MMP-13 in cultured chondrocytes [22]. Our results suggest that Pro-hyp may regulate chondrogenic differentiation and contribute to skeletal development in chondrocytes under hypoxic conditions via elevated Mmp13 mRNA expression. 

### 2.3. Pro-Hyp Further Increases Sox9 Protein Level without Affecting Runx2 Protein Level under Hypoxic Conditions Compared to Normoxic Conditions

**Figure 3:**
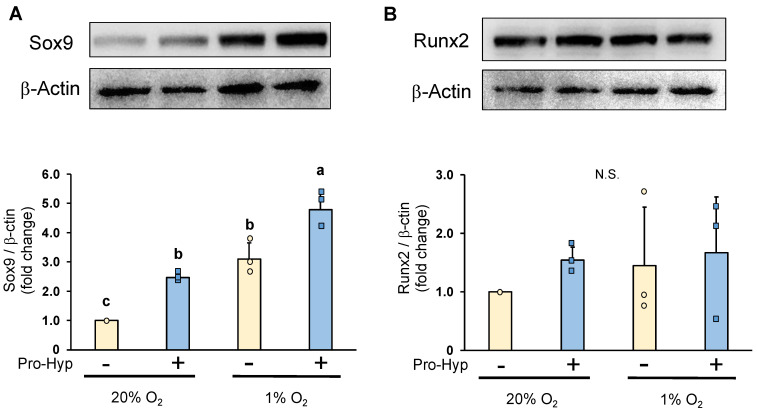
ATDC5 cells were seeded in a 60 mm dish at a density of 1.8 × 10^5^ cells/well and cultured in either normoxic condition (20% oxygen) or hypoxic condition (1% oxygen) for four days. The cells were treated with or without Pro-Hyp (0.1 mmol/L) for 24 h before harvest. Western blotting imaging and densitometric analysis of the expressions of (**A**) Sox9, and (**B**) Runx2 in ATDC5 cells. Data are expressed as means ± SD. Dots represent individual samples. Different letters above the boxes indicate significant differences. *p* < 0.05. N.S.: no significant differences.

Under normoxic conditions, chondrocytes cultured in a Pro-Hyp-containing medium exhibited a significant increase in Sox9 protein levels when compared to the control (Figure 3A). Subsequently, we examined the impact of low oxygen levels on Sox9 expression and observed an elevation in Sox9 protein levels. Notably, the addition of Pro-Hyp to the culture medium further potentiated the increase in Sox9 expression under hypoxic conditions. Conversely, neither normoxic nor hypoxic conditions showed any alteration in Runx2 protein expression. Furthermore, the addition of Pro-Hyp had little effect on Runx2 levels (Figure 3B). The results obtained through Western blot analysis demonstrated that Pro-Hyp strongly upregulates Sox9 expression without affecting Runx2 expression during the early differentiation of chondrocytes under hypoxic conditions. 

Overall, Pro-Hyp is capable of regulating chondrocyte differentiation via a mechanism involving Sox9 upregulation in hypoxic conditions. Our findings advance our understanding of the regulatory mechanisms of chondrocyte differentiation and highlight the potential therapeutic significance of Pro-Hyp in cell-based therapy for degenerative joint diseases, such as osteoarthritis.

## 3. Materials and Methods

### 3.1. Chemicals

Pro-Hyp was purchased from Bachem (Bubendorf, Switzerland). Fetal bovine serum (FBS) was purchased from Sigma (St. Louis, MO, USA). 

### 3.2. Cell Culture and Treatment

Mouse chondrogenic ATDC5 cell line was obtained from the RIKEN cell Bank (Tsukuba, Japan) and was cultured in DMEM/F-12 (Gibco, Thermo Fisher Scientific, Inc., Waltham, MA, USA) with 10% fetal bovine serum and 100 U/mL penicillin. The cells were incubated in a wet incubator with 5% CO_2_ and 95% air at 37 °C for normoxic conditions and in a hypoxic incubator (SMA-30D; Astec, Fukuoka, Japan) with 5% CO_2_ and 1% O_2_ balanced with N_2_ at 37 °C for hypoxic conditions.

### 3.3. Cell Proliferation

Cell proliferation was determined using the WST-1 assay (Cell Counting Kit; Dojindo Laboratories, Kumamoto, Japan). Plates were read using a microplate reader (Perkin Elmer, Inc., Waltham, MA, USA) at a wavelength of 450 nm.

### 3.4. Alcian Blue Staining

The cells were fixed with 20% formalin at room temperature for 20 min. The fixed cells were then washed three times with deionized water and twice with 3% acetic acid. Alcian blue (pH 1.0, Muto pure chemicals, Japan) was added for 2 h, and the cells were washed with deionized water. The Alcian blue stained areas were scanned using an image scanner and analyzed qualitatively using Image J software version 1.53k.

### 3.5. RNA Preparation and Quantitative RT-PCR (qPCR)

Total RNA was extracted from the cells using the RNeasy Mini Kit (Qiagen, Germany), and cDNA was synthesized from 5 mg of mRNA using the Prime ScriptTM Reagent Kit (Takara Bio Japan, Japan). qPCR was performed by TB Green^®^ Fast qPCR Mix (Takara). b-actin was used as the internal control for normalizing the expression of target genes. The primers used are listed in Table 1.

### 3.6. Western Blot Assay

Cells were washed twice with ice-cold PBS and then lysed with RIPA buffer consisting of 25 mM Tris-HCl (pH 7.6), 150 mM NaCl, 1% NP-40, 1% sodium deoxycholate and 0.1% SDS and containing a protease inhibitor cocktail. Cell lysates were centrifuged at 15,000 rpm for 20 min, and the supernatants were collected as protein samples. The protein concentration of each sample was measured with BCA Protein Assay Reagent (Thermo Fisher Scientific). Proteins were separated by SDS-PAGE and transferred to polyvinylidene difluoride (PVDF) membranes using Trans-Blot^®^ Turbo™ Transfer System (Bio-Rad Laboratories). After blocking with 5% skim milk in TBS-T consisting of 10 mM Tris-HCl (pH 7.4), 1.37 M NaCl, and 0.1% Tween 20 for 30 min at room temperature, the membranes were incubated with rabbit anti-Sox9 (catalog #36169; Cell Signaling Technology, Danvers, MA, USA), rabbit anti-Runx2 (4835; Cell Signaling Technology, Danvers, MA, USA), or rabbit anti-β-actin (3700; Cell Signaling Technology) 1/1000 diluted in a blocking buffer for 1 h at room temperature. The membranes were washed with TBS-T and then incubated for 45 min at room temperature with an HRP-conjugated rabbit anti-mouse IgG (7074; Cell Signaling Technology, Danvers, MA, USA) 1/2000 diluted in TBS-T. Labeled proteins were detected with EZ west Lumi plus (ATTO). Band intensities were determined using ImageJ Software.

### 3.7. Statistical Analysis

Data are expressed as means ± SD. Statistical analyses were performed using SPSS Statistics for Mac, Version 25.0 (IBM Corp., Armonk, NY, USA). Differences among multiple groups were compared using one-way analyses of variance with Tukey post hoc tests. Values with *p* < 0.05 were considered statistically significant. Dots represent individual samples.

## 4. Conclusions

In this study, we show for the first time that Pro-Hyp strongly promotes the early differentiation of chondrocytes under physiological hypoxic conditions. It has been reported in bone tissue that demineralized collagen produced by osteoclasts is likely a signal that attracts osteoblasts, which are adjacent to bone remodeling sites [23]. Degradation products of type 2 collagen are also reported to be directly involved in regulating cartilage homeostasis, possibly by increasing gelatinase activity and matrix degradation [24]. The current results indicate that Pro-Hyp, a bioactive peptide produced during collagen metabolism, may function as a remodeling factor or ECM remodeling signal that regulates chondrocyte differentiation in physiological hypoxic conditions. Overall, this study highlights the potential of Pro-Hyp as a therapeutic target for promoting chondrocyte differentiation in cartilage tissue engineering and regenerative medicine applications.

## Figures and Tables

**Table 1:** Sequences of primers used in qRT-PCR.

Gene	Forward Sequence (5′-3′)	Reverse Sequence (5′-3′)
Sox9	CCAGCAAGAACAAGCCACAC	GCTCAGTTCACCGATGTCCA
Col2α1	AGGTGCTCAAGGTTCTCGTG	GCTCCAGGAAGACCAGGTTC
Aggrecan	CCAAACCAGCCTGACAACTT	TCTAGCATGCTCCACCACTG
Runx2	TAAGAAGAGCCAGGAGGTGC	AGGTACGTGTGGTAGTGAGTG
MMP9	TGAATCAGCTGGCTTTTGTG	GTGGATAGCTCGGTGGTGTT
MMP13	AGGCCTTCAGAAAAGCCTTC	TCCTTGGAGTGATCCAGACC
β-actin	AAGGCCAACCGTGAAAAGAT	GTGGTACGACCAGAGGCATAC

## Data Availability

All relevant data are within the paper.
